# Representation of Friendship and Aggressive Behavior in Primary School Children

**DOI:** 10.3389/fpsyg.2022.835672

**Published:** 2022-04-19

**Authors:** Anna Di Norcia, Anna Silvia Bombi, Giuliana Pinto, Chiara Mascaro, Eleonora Cannoni

**Affiliations:** ^1^Department of Developmental and Social Psychology, Sapienza University of Rome, Rome, Italy; ^2^Department of Education, Languages, Intercultures, Literatures and Psychology, University of Florence, Florence, Italy

**Keywords:** friendship quality, aggressive behavior, children’s gender, pictorial representation, middle childhood

## Abstract

This study examines the representation of friendship during middle childhood and its impact on aggressive behavior. The literature shows that friendship is almost a “gym of social skills,” which, in turn, are protective factors against aggressive behavior; in this regard, the quality of friendship is especially important, but this quality becomes less and less accessible to direct observation as children grow older and spend most of their time in the externally regulated environment of primary school. To assess friendship quality requires allowing children to present their own perspective on the relationship, a goal that we have tackled through drawing. Children aged 6–11 years were individually asked to draw themselves and a close friend in two situations (i.e., relational wellbeing and relational distress) and to complete a 20-item scale of physical and verbal aggression. Data were analyzed with three main aims, namely, (1) to show if and how the representation of two core features of relationships (i.e., relatedness and individuality) changes according to the situation and/or according to the children’s gender; (2) to focus on the representation of distressing situations to verify if they coincide with forms of conflict and if they differ according to the children’s gender; and (3) to verify if the strength of indices of relatedness and individuality, both in situations of wellbeing and distress, predicts children’s tendency to enact aggressive behaviors. The results confirm that relatedness is the dominant feature of friendship, especially in the situation of wellbeing and when the situation becomes distressing. Conflict is not always present when children do not feel fine with their friends; boys and girls do not differ significantly in this regard, but they do differ in terms of the management of relatedness and individuality when problematic situations arise. In line with previous studies, sex is the main predictor of aggressive behavior with peers, with boys more at risk than girls; however, the capacity to relate with one’s own friend even in difficult times (in which boys are not inferior to girls) predicts lesser aggression with peers in general.

## Introduction

### Social Competence and Friendship

This study has been conducted in the framework of research about the protective role that friendship, as an arena for developing social competence, plays against aggression in middle childhood. Social competence has been defined as the capacity to adapt to various situations in ways that are satisfying for the individual but are also accepted by their partners (Rose-Krasnor, 1997), and many studies demonstrate its negative correlation with behavioral problems (Huber et al., 2019; Hukkelberg et al., 2019). Since social competence is *cross-sectional* and *transactional* in its nature, different experiences with both adults and peers are required for its acquisition (Milligan et al., 2017). Precisely for this complexity, the molecular components of social competence are difficult to enlist, as various authors have repeatedly observed in the last 40 years (Waters and Sroufe, 1983; Cavell, 1990; DuBois and Felner, 1996; Topping et al., 2000; Cillessen and Bellmore, 2011). However, there is a large agreement about the developmental nature of social competence and about the importance of relevant experiences at appropriate ages to build it. An effort to specify this experiential timing has been carried out recently by Junge et al. (2020). Based on a model originally proposed for preschoolers by Rose-Krasnor and Denham (2009) and supported by rich empirical data, these authors traced a series of steps from infancy to adolescence, outlining the skills most necessary according to the children’s age and contexts. While child-adult relationships are crucial in infancy, at the onset of childhood, new challenges are posed by interactions with peers; subsequently, in the primary school context, gaining peer acceptance and developing more stable and intimate friendships become important, setting the stage for the most complex social tasks of adolescence (Junge et al., 2020). Middle childhood is also a period in which aggressive behavior, normative to some extent in preschoolers, declines, because of the increasing ability to regulate emotions and control one’s own behavior (Tremblay et al., 2017); these skills become more important to avoid rejection and maintain friendly relationships, goals that are equally important for boys and girls of this age, even if addressed with different strategies, as we will discuss in the following sections (Underwood et al., 2006).

Friendship is undoubtedly important *per se*, being central to our lives at every age, and its many facets have been studied by philosophers (Helm, 2021); social, cross-cultural, and developmental psychologists (Harré and Moghaddam, 2014; Wrzus and Neyer, 2016; Lu et al., 2021); and sociologists (Allan, 2022) and cultural anthropologists (Hruschka, 2010). From their distinct approaches, we know that some features of friendship vary according to the partners’ environment and their personal characteristics; however, the voluntary character and reciprocal concern on the part of each friend for the welfare of the other are unanimously recognized as constitutive properties.

To maintain this reciprocity and personal satisfaction, a balance is required between relatedness and individuality (Krenz et al., 2021). Relatedness (and the germane concepts of affiliation, connectedness, bonding, or simply psychological proximity) and individuality (and the germane concepts of agency, exploration, and autonomy) are considered universal ingredients of social relationships, even if in diverse proportion according to culture, type of relationship, and circumstances (Guisinger and Blatt, 1994; Rothbaum and Trommsdorff, 2007). Also as suggested by theorists of child relationships (Clark and Ladd, 2000; Neff and Harter, 2003), relatedness and individuality are not opposite poles of a single dimension, but rather components that vary to some degree in any relationship, friendship included; however, a certain degree of relatedness must always be there, otherwise the relationship dissolves.

This implies that some competence in interpersonal exchanges is necessary to keep a friendship alive even in face of occasional disagreements or other relational difficulties (Erdley et al., 2001; Gifford-Smith and Brownell, 2003). The context of primary school and the notable cognitive development that takes place during middle childhood make this period especially important for friendship development (Miller et al., 2020). A qualitative study by Walker et al. (2016) found that 9-year-olds were able to describe the virtues needed to qualify a friendship as “real” vs. “fake,” including commitment, loyalty, forgiveness, and many others. Adopting a friendship quality scale, Maunder and Monks (2019) found that since the age of 7 years, children with high scores of companionship, help, security, closeness, and low scores of conflict had more lasting relationships with their best friends. A good friendship is the result of social competence but also provides an opportunity to improve it: For example, a longitudinal study conducted by Glick and Rose (2011) showed that the quality of friendship (including strategies of conflict solution) predicted an increase of competent social responses from middle childhood to early adolescence more than the sheer number of friends. Finally, children from environments as different as the United States and China recognize friendship as a factor of wellbeing and resilience in their primary school experience (Ni et al., 2018).

### Conflict, Aggression, and Gender Differences

In their manual about *Interpersonal Conflict*, Hocker et al. (2022, p. 3) present conflict as “an expressed struggle between at least two interdependent parties who perceive incompatible goals, scarce resources, and interference from others in achieving their goals.” As such, conflict does not imply aggression in itself but easily lends to it if not appropriately managed, as many studies have demonstrated (Forgas et al., 2011). Friendship is not immune to conflict, since partner discrepant goals or expectations may lead to mild opposition or even to aggressive confrontation; sometimes, difficulties lead to moving away, momentarily suspending the relationship. In general, a certain degree of aggressiveness is quite common in preschoolers but decreases at the time of school entry; continuing aggressive behavior is a source of risk more or less severe, according to the frequency and severity of aggressive acts (Campbell et al., 2006). During middle childhood, many children develop the ability to cope with provocation and to dissimulate anger (Salvas et al., 2011), which allows them to avoid aggressive conflicts and maintain their friendships. A study of aggressive children shows that they have difficulty tolerating provocations from unfamiliar peers, but they also mismanage conflicts with best friends, interrupting interaction rather than resolving the ongoing conflict (Burgess et al., 2006). This can have long-lasting effects, as shown by a retrospective study carried out with about a thousand young adults (King et al., 2017): The authors found a correlation of adult aggressive behavior not only with the difficulty of maintaining their current friendships but also with the poor quality of the friendships experienced during childhood.

Boys and girls are likely to manage difficult times in friendship differently. Boys are notoriously more inclined than girls to open aggression, physical and verbal, while relational aggression (e.g., gossip or exclusion) is more frequent for girls (Card et al., 2008). In the context of friendship, difficulties are more likely to result in direct confrontation and even aggressive acts for boys than for girls, who are instead more prone to try compromise solutions; for these reasons, girls are often considered more competent than boys in maintaining their friendships, at least during middle childhood (Xu et al., 2020). In one of the studies based on young children’s self-report, Murphy and Eisenberg (2002) described some frequent reasons of conflict with friends and non-friends in children from 7 to 11 years of age: physical offense (not necessarily intentional); damaging or taking another’s object; trying to impose oneself on the partner; ignoring him/her; breaking a rule or lying; and verbally offending. Physical harm as a cause of the conflict was mentioned more often by boys; girls described more constructive behaviors aimed at conflict solution. These results are in line with the higher frequency of boys’ physical confrontations, and the superior relational competence attributed to girls in the field of peer relationships (Rose and Rudolph, 2006). However, a more recent work by MacEvoy and Asher (2012) has challenged this view, presenting data about stronger reactions of girls to hypothetical situations in which a friend violated friendship expectations. Therefore, if the inappropriate actions of the partner go, so to speak, at the heart of the relationship, girls do not seem so good at negotiating constructive solutions.

In any event, a good friendship should survive occasional difficulties, and the ability to maintain the relationship alive is a crucial social competence. A longitudinal study demonstrated that socially inappropriate behaviors make it difficult for children to sustain lasting friendships (Murray, 2012). Complementing this finding, another longitudinal study with fifth graders showed that, controlling Time 1 aggression, boys who lost a friendship and were unable to replace it became significantly more aggressive than boys who had a best friend at both Times 1 and 2 (Wojslawowicz Bowker et al., 2006). These studies exemplify how the ability to manage interpersonal exchanges without resorting to aggression, even in case of disagreement or conflict, is a very important component of social competence (Milligan et al., 2017) and point to the importance of documenting the presence of this ability even at the beginning of school age.

### Methodological Problems in the Study of Friendship in Middle Childhood

The quality of friendship is not equally easy to examine across ages. Similar to any other close relationship, children’s friendship is made up of both actions and thoughts. As leading scholars such as Kelley (1984), Berscheid (1985), and Hinde (1987)—to cite a few—already suggested more than 30 years ago, the interactions are only the building blocks of relationships: What partners do at any given moment is always a function of their memory of previous interactions and expectations for future ones. This focus on internal factors still remains the basis for the *science of relationships* (Regan, 2011) and continues to produce a methodological problem from a developmental perspective.

Young children’s interpersonal behavior is sufficiently “transparent” that visible exchanges between children who declare a mutual preference would allow observers to understand a lot about their friendship; however, this is no longer true as children grow older. In fact, friendship functions change, passing from kindergartners’ coordinated play to the appreciation of personal qualities and shared norms in middle childhood and reaching that adolescents’ search for intimacy and “mirroring into each other” that Sullivan first proposed as the friendship benchmark (Sullivan, 1953); accordingly, relevant features of friendship become less obvious “from outside.”

Besides these changes, the context of primary school offers much less opportunities to observe free interactions than preschool and kindergarten. Thus, during early childhood, behavioral observation can be the choice method, from the age of 6 or 7 years, but now it becomes essential to question the protagonists themselves about their relational experiences. Since the convenient format of written questionnaires would be unsuitable for children at their early steps of schooling, one way to access their ideas is the oral interview, a method expensive in terms of time and difficulty. Moreover, as some classical studies have shown (Selman, 1980; Youniss, 1980) and recent inquiries have confirmed (Marcone and Caputo, 2019), the explicit conceptualization of relationships, including friendship, is still “in progress” at this age, somewhat obscuring the tacit knowledge that guides children’s interpersonal behavior.

### Giving Voice to Children by Means of Drawing

This methodological difficulty is perhaps one of the reasons why research, after concentrating on younger children at its beginnings (Bukowski et al., 1997), has focused more on preadolescents and adolescents, with whom it is easier to use structured verbal tools. A fairly recent review (Crowe et al., 2011) examined the instrument for studying social functioning published over a period of 20 years; although the scope of the review was broader (including not only relationships but also interactions, emotions, and personal characteristics), only a dozen of the 86 instruments reviewed were aimed at first or second graders; moreover, the few instruments focused on friendship quality were suggested for use not earlier than third grade (Parker and Asher, 1993; Grotpeter and Crick, 1996) or fifth grade (Bukowski et al., 1994). Yet, children’s voice is important, especially when problematic aspects of a personal relationship are studied: In fact, an effective children’s guidance to manage such difficulties requires as its basic feature a genuine understanding of the child’s perspective (Gartrell, 2017).

Drawing is precisely a way to give voice to children, even at a relatively early age; it provides an alternative form of representation, tapping into visual and emotional meanings; it leads to a succinct presentation of the key elements of participants’ experiences, and, last but not least, “it allows participants’ unique experiences, rather than researcher constructs, to be communicated” (Freeman and Mathison, 2009, p. 114). This is especially important when you want to question a school-age child, whose negative experiences occur more and more farther from the direct gaze of adults and on which, therefore, it is difficult to ask “the right questions.” Examples of the use of pictorial representations of relationships (in this case, family) with children of this age are Carlson et al. (2004) and, more recently, Pace et al. (2020); refer to also Pace et al. (2021) for a useful review and discussion of this pictorial approach.

The interpretation of drawings collected outside a clinical setting has been often criticized for the risk of misinterpretation (e.g., Joiner and Schmidt, 1997). The method adopted here, i.e., PAIR (Pictorial Assessment of Interpersonal Relationship; Bombi et al., 2007), was developed precisely to overcome the limits of subjective interpretations. The way in which drawings are collected for PAIR stresses the adult’s need to know something from the child (friendship, in this case), which can be shown through a drawing. The scales for coding the drawings are based on several studies designed to test their validity (summarized in Bombi et al., 2007) and provide information about several distinct features of dyadic interpersonal relationships, including relatedness, individuality, and conflict management, whose importance in friendship has been discussed above. PAIR has been applied to a variety of children’s relationships by our research group (Lecce and Pinto, 2004; Pinto and Bombi, 2008; Laghi et al., 2014; Cannoni and Bombi, 2016) and by other independent researchers (Misailidi et al., 2012; Rabaglietti et al., 2012; Sándor et al., 2012; Guidotti et al., 2020). A further strength of PAIR is the use of two drawings for each participant, a manageable task even for young children, and useful for the researcher to keep under control any pictorial idiosyncrasies, not to be interpreted as indicative of ideas on the theme drawn.

### Aims and Hypotheses

Based on the literature summarized above, this study aims:

(1) To examine how girls and boys depict themselves and a friend in two opposite situations, namely, wellbeing and distress. Children are expected, independently from gender, to show more relatedness than individuality in both drawings, to communicate the existence of the friendship between the depicted characters. However, the balance between these components of the relationship should be altered in the representation of distress, with a loss of relatedness and an increase of individuality that reflects the lesser harmony implicit in any distressing situation or an increase of relatedness with negative valence (i.e., approaching the partner to hit them; talking to insult); in the light of literature quoted above, these different ways of representing a distressing situation are likely to characterize, respectively, girls’ and boys’ drawings.

(2) To verify how often, for boys and girls, distress is perceived as conflictual and in which form. The request of representing a distressing situation is an open task, in which children cannot resort to those scenarios of happy play, conversation, and exchange of affect that commonly portray friendship. Instead, they have to select the specific situations that hinders the wellbeing of themselves, of their friend, or both; these can be real instances of what happens in their daily lives, or examples of what they fear most, and which perhaps they have experienced only a few times. In short, they must choose to show the adult what they consider most destructive for wellbeing in a friendly relationship. Again, in light of the studies presented above, we expected that boys and girls will present different types of distressing situations, even if it seems not possible to advance more precise hypotheses due to the novelty of our approach.

(3) To investigate if relatedness and individuality in wellbeing and distress predict aggressive behaviors in peer relationships (i.e., outside the friend’s dyad). We expected that, in line with most studies, the male gender would predict higher aggressiveness and the representation of friendship should add information in this regard. In particular, given the importance of friendship quality as a protective factor, it is possible that a representation of strong relatedness and reduced individuality in each situation would predict lesser aggressiveness with other peers; or it is possible that only the ability to maintain relatedness in distressing situations, without stressing one’s own individuality, would constitute a predictor. In the absence of studies specifically based on pictorial representation, we will test both hypotheses.

## Materials and Methods

### Participants and Procedure

Participants were 133 primary school children, recruited through convenience sampling based on the school’s willingness to participate in the study. They were 64 boys and 69 girls, aged 6–11 years (*M*_age_ = 8.6; SD_age_ = 1.12). Data were collected in central Italy. The educational level of mothers who provided the required demographic information (70% of participants) was as follows: 5% only grade school, 38.3% only high school degree, and 56.7% college degree.

Data of this study came from a broad research project on the social and emotional competence of children in Italian primary schools. Only the measures considered in this study were described. A questionnaire about demographic information was completed by parents, after accepting informed consent ensuring the voluntariness and anonymity of their participation and participation of their children. Children too orally accepted informed consent and completed drawings and a questionnaire about physical and verbal aggression. This research and its procedure were approved by the ethics committee of [*blinded for peer review*].

### Measures

#### Individual Information

Parents reported the gender (0 = girl; 1 = boy) and age of the son/daughter about whom they were completing the questionnaire and information about their own educational level.

#### Children’s Representation of Friendship

Children’s representation of friendship was assessed through drawings. Each child was given a white sheet of 8 1/2 × 11 in. and a pencil and was required to draw themselves with a friend in two circumstances, namely, when “things go well, you feel fine together, you get along well” (wellbeing) and when “things are not going well, you don’t [*sic*] feel fine together, you don’t [*sic*] get along” (distress). No time limits were assigned, but children completed the drawing in 20’ as a maximum.

Three of the scales that make up the abovementioned PAIR instrument (Bombi et al., 2007) were used to score the drawings, namely, cohesion, distancing, and conflict. The scales of cohesion and distancing separately measure two constitutive elements of the relationship, i.e., the number of indices of relatedness on the one hand and the number of indices of individuality on the other hand. Each scale includes six subscales, to be scored dichotomously (0 = absence; 1 = presence of one or more pictorial indices), pertaining to various aspects of the represented interactions (such as looking to each other or looking away) and the spatial distribution of the figures (such as inclusion in the same area or separate areas of the depicted scene). Cohesion and distancing are not the poles of a continuum, as their indices can coexist in the same drawing (e.g., figures can look at each other, while being in separate spaces).

It is important to note that the indices of cohesion can be employed to represent interactions with different meanings: caressing or hitting as it often happens between siblings (Lecce et al., 2002), praising or reproaching as we can see in educational relationships (Bombi et al., 2020), and so on. In fact, aggression and discord are ways to interact that in long term can destroy a relationship but are not immediate instances of bond dissolution.

The third PAIR scale employed in this study, conflict, is precisely a classification of negative interactions that can occur in a relational history and compacts in three categories the instances described by Murphy and Eisenberg (2002): (1) Opposition (i.e., disputes arising from objects property or discrepant wills); (2) aggression (i.e., physical or verbal offenses); (3) interactions break (i.e., ignoring the partner or showing a desire of interrupting the interactions). Drawings in which none of these negative behaviors appears are considered 0) no conflict, even if signs of distress can appear (such as indices of negative emotions, which are examined in another PAIR scale, emotions).

Each drawing was rated by two independent judges, who had not participated in the data collection and were blind to the aims of the study. The two judges reached a significant level of interreliability for the three scales (correlation coefficients: 0.86; 0.91; and 0.87 with *p* < 0.001). For the final score assignment, they discussed each score on which they disagreed until a full agreement had been reached.

#### Physical and Verbal Aggression

Children were asked to complete a scale of physical and verbal aggression (Caprara and Pastorelli, 1993); younger children who encountered difficulties in reading and/or writing were helped by a research assistant. The questionnaire included 20 items describing aggressive behaviors (e.g., I happen to quarrel with other children; sometimes I tell lies), with a 3-point response scale as follows: 0 = never or almost never; 1 = sometimes; and 2 = often. The total score was calculated as a mean of the single score item. The alpha reliability index in this sample was 0.82.

### Data Analyses

Data analyses were performed using the statistical program SPSS version 25.0. Descriptive statistics and bivariate Pearson’s and Kendall Tau-b correlations were computed on the study variables. Two repeated-measures analyses of variance (ANOVAs) were performed, namely, the first on cohesion and distancing in each drawing (wellbeing and distress) as within-subjects factors and gender as the between-subjects factor; the second, on drawing of distress only, on cohesion and distancing as within-subjects factors, and conflict categories as the between-subjects factor. *Post hoc* analyses were carried out, when necessary, with Tukey’s test or with a *t*-test for repeated measures. Frequencies of categories in the conflict scale were compared by gender through the chi-square test. Finally, a hierarchical regression analysis was conducted, to investigate the predictors of physical and verbal aggression among the variables measured through children’s drawings. In the first step, sex and age were entered; in the second step, cohesion and distancing in wellbeing drawings were added; in the third step, cohesion and distancing in distress drawings were added to the regression equation.

## Results

Descriptive statistics and bivariate Pearson’s correlations are reported in Table 1.

**TABLE 1:** Descriptive statistics and bivariate Pearson’s correlations on study variables.

	1	2	3	4	5	6	7	8	Range	*M* (*SD*) boys	*M* (*SD*) girls	*M* (*SD*) total
1. Gender (1 = boys; 2 = girls)	1								−	−	−	−
2. Age	0.08	1							6–11			8.6 (1.12)
3. Wellness-cohesion	0.04	0.12	1						0–6	1.83 (1.16)	1.92 (1.45)	1.87 (1.30)
4. Wellness-distancing	0.17*	0.07	0.04	1					0.6	1.14 (1.22)	0.78 (0.87)	0.97 (1.08)
5. Distress-cohesion	−0.12	0.06	0.34**	0.14	1				0–6	1.72 (1.14)	1.44 (1.32)	1.59 (1.23)
6. Distress-distancing	0.20*	0.11*	0.23**	0.15	−0.24	1			0–6	1.00 (0.89)	1.45 (1.31)	1.22 (1.13)
7. Physical and verbal aggression	0.27*	0.15	−0.16	−0.07	−0.02	0.12	1		0-2	0.53 (0.40)	0.34 (0.27)	0.44 (0.35)
8. Conflict	0.01	−14	0.25**	0.15	0.10	0.20	0.01	1	0.3			

*p < 0.05, **p < 0.01.

**FIGURE 1:**
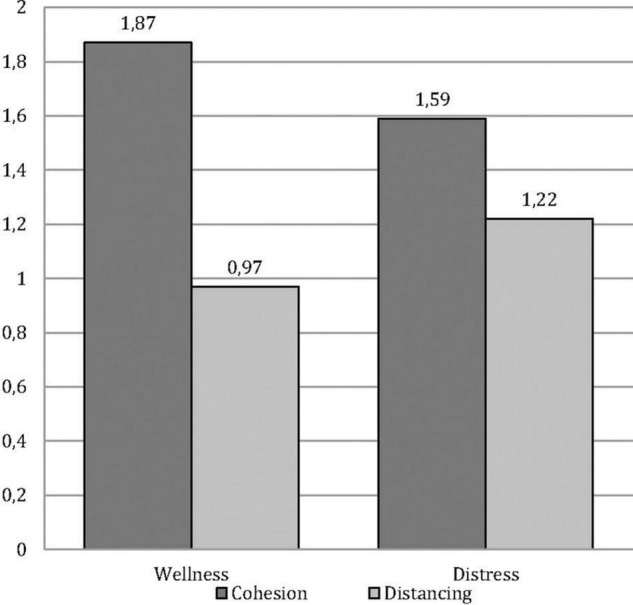
Cohesion and distancing by wellness and distress.

**FIGURE 2:**
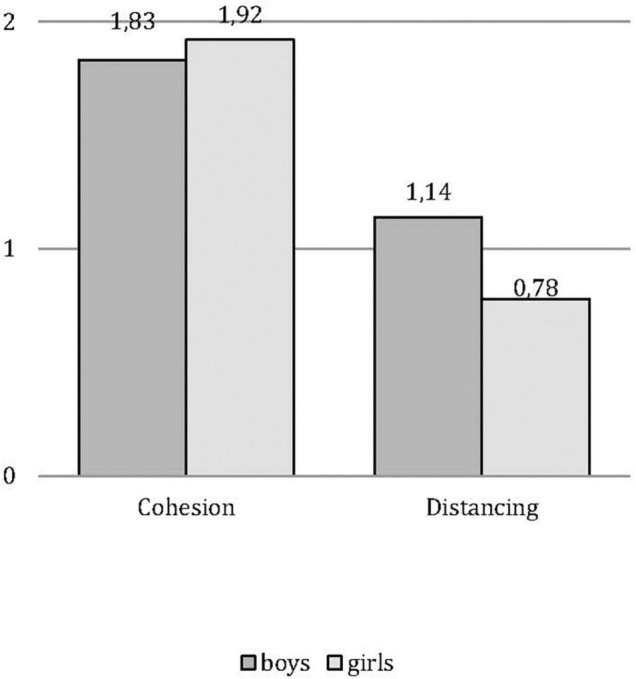
Cohesion and distancing in wellbeing by gender.

**FIGURE 3:**
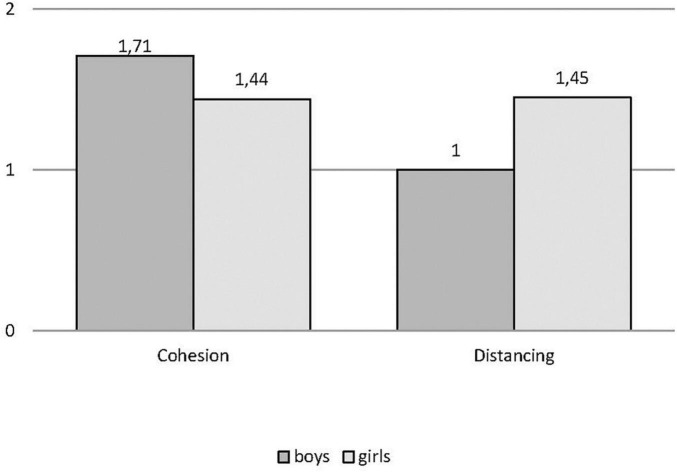
Cohesion and distancing in distress by gender.

The first repeated-measures ANOVA examined cohesion and distancing in each drawing (i.e., wellbeing and distress) by gender. Findings showed a significant main effect of cohesion (1.73) over distancing (1.09) [*F*_(1, 129)_ = 31.78; *p* < 0.000; η^2^_*partial*_ = 0.20] and two significant interactions, namely, cohesion and distancing by drawings of wellbeing and distress [*F*_(1, 129)_ = 7.24; *p* = 0.008; η^2^_*partial*_ = 0.05], as shown in Figure 1, and cohesion and distancing in drawings of wellbeing vs. distress by gender [*F*_(1, 129)_ = 8.12, *p* = 0.005; η^2^_*partial*_ = 0.06], as shown (separately for clarity) in Figures 2, 3. Specifically, the *post hoc* comparisons on the first interaction showed that cohesion scores were higher than distancing in both drawings of wellbeing (*p* < 0.001) and distress (*p* = 0.024); if compared across drawings, cohesion decreased significantly from wellbeing to distress (*p* = 0.026) while distancing increased (*p* = 0.049). The *post hoc* comparison on the second interaction showed that in the drawings of wellbeing, cohesion is higher than distancing for boys (*p* = 0.001) and girls alike (*p* < 0.001), while in the drawings of distress, this difference in favor of cohesion remains only for boys (*p* < 0.001), while girls introduce an equal amount of indices of cohesion and distancing (0.953); comparing cohesion and distancing by gender in each drawing, no significant difference appears, except for distress drawings, in which the amount of distancing is significantly higher for girls (*p* = 0.02).

The analysis of conflict, carried out on the distress drawings, showed that the frequencies of the four categories were significantly different (χ^2^_3_ = 31.24; *p* < 0.001): About half of the participants (*N* = 60; 32 boys, and 28 girls) represented the distressing situation without indices of conflict; indices of aggression were represented in 31 drawings (i.e., 21 boys and 10 girls) followed by the opposition (*N* = 24; 9 boys and 15 girls) and finally by interactions break (*N* = 18; 7 boys, 11 girls). The difference of frequencies for boys and girls does not reach statistical significance (χ^2^*p* = 0.09).

**FIGURE 4:**
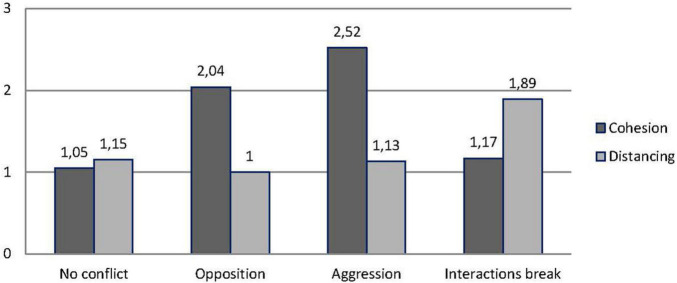
Cohesion and distancing by conflict categories.

The second repeated-measures ANOVA, performed only on the distress drawings, compared cohesion and distancing by categories of conflict. Findings showed main effects of the repeated measures [cohesion = 1,59 vs. distancing = 1,22; *F*_(1, 129)_ = 6,01; *p* = 0.016; η^2^_*partial*_ = 0.04] and of conflict categories (more indices of cohesion + distancing in aggression (3,6) than in no conflict (2,2) with intermediate scores (3,04 each) for opposition and interactions break; *F*_(3, 129)_ = 8.49; *p* < 0.001; η^2^_*partial*_ = 0.16] and an interaction between repeated measures and the conflict categories, shown in Figure 4 [*F*_(3, 129)_ = 8.8; *p* < 0.001; η^2^_*partial*_ = 0.17].

The hierarchical regression analysis conducted to investigate the predictors of physical and verbal aggression among the variables measured through children’s drawings showed the following findings. Step 1 was significant and explained the 0.8% of the variance in physical and verbal aggression, with female sex predicting significantly lesser aggression; step 2 did not add a significant increase to the explained variance; step 3 was significant explaining the 0.17% of the variance with a significant *R*^2^ increasing (*p* = 0.01): both sex (β = −0.22; *p* < 0.05) and cohesion in distress situations (β = −0.33; *p* = 0.01) were significant negative predictors (refer to Table 2).

**TABLE 2:** Summary of hierarchical regressions predicting physical and verbal aggression from drawing variables.

					Physical and verbal aggression								
	Step 1				Step 2				Step 3				
Predictors	B	SE B	β	R2	B	SE B	β	R2	B	SE B	β	*R*2
				0.08**				0.09				0.17**
Sex (*M* = 1; *F* = 2)	−0.16	0.06	−0.24*		−0.15	0.07	−0.22*		−0.15	0.07	−0.22*	
Age	0.05	0.03	0.17		0.05	0.03	0.17		0.05	0.03	0.18	
Wellness cohesion					−0.01	0.03	−0.03		0.03	0.03	0.11	
Wellness distancing					0.03	0.03	0.10		0.05	0.03	0.15	
Distress cohesion									−0.09	0.03	−0.33**	
Distress distancing									−0.04	0.03	−0.12	

**p ≤ 0.01; *p < 0.05.

## Discussion

This research contributed to our knowledge of the characteristics of friendship in middle childhood as an interpersonal bond that coexists with individual autonomy. In particular, our data (1) enhance our understanding of friendship representation in middle childhood, having reached students of all grades of primary school; (2) provide a fresh perspective on difficulties in the interactions between friends, due to the choice of an open-ended task such as drawing oneself with a friend in two different situations, namely, wellness and distress; and (3) demonstrate that the ability to maintain a bond with the friend, even if connoted with opposition or even aggression, is crucial as a protective factor for a widespread enactment of aggressive behavior with peers.

The dimensions of relatedness and individuality, as measured by indices of cohesion and distancing, appear (as in previous studies with drawing in which positive and negative situations were compared; Lecce et al., 2002; Bombi et al., 2020; Guidotti et al., 2020) very revealing of the relational quality, since wellbeing and distress with one’s own friend are reflected in the relative amounts of each dimension. In friendship, a relationship not prescribed by kinship or role, creating a sense of relatedness is a necessary condition of existence; interpersonal difficulties can diminish this sense of relatedness and increase the need for individual autonomy, but these alterations should not bring to the disruption of the relationship as it would happen if cohesion indices would be minimized. Boys and girls do not differ in the maintenance of sufficient cohesion, but girls appear to perceive difficulties in terms of an increased affirmation of independence more than boys. These results speak for different ways of managing difficulties, more than a superior social competence of girls (MacEvoy and Asher, 2012).

The data about conflict categories confirm that the types of distressing situations depicted by children are similar to those described verbally (Murphy and Eisenberg, 2002); however, the difference by gender that appeared from the analysis of cohesion and distancing does not reach significance in terms of content analysis with the conflict scale. It was also quite surprising to find that almost half of the participants did not conceive the distressing situation in terms of conflict. A qualitative exam of these drawings shows that in the large majority of cases, children did introduce signs of distress, based on the depiction of facial emotions or verbalizations making reference to external causes of worry or sadness, such as not being able to play together because of an illness or punishment: All these aspects could be the object of further studies. These considerations permit us to consider the absence of conflict in children’s drawings as a conceptual choice and not as a simple consequence of pictorial limitations: In fact, representing sad or angry faces is not easier than showing indices of proximity or distance between the figures, and it requires a similar capacity to write in a balloon or “We cannot play today” instead of “Give me your play station!” or “I’m really offended.”

The concentration of drawings in the category of no conflict, and the small number of boys in the categories of opposition and interactions break, has prevented gender from being included as an independent variable alongside conflict categories. However, this analysis provided important information about the possible impact of children’s representation of opposition, aggression, and interactions break on the friendship maintenance. In this study, it is important to remember that children were not asked to report the frequency of conflict but to present (pictorially) what they consider a situation that causes wellbeing or distress with their friends. So it is not surprising that quarrels, aggressive acts, or momentary withdrawal from interactions are presented as detrimental for interactional wellbeing. But why does cohesion remain high in the two cases of opposition and aggression? Cohesion, as we have said above, includes any act that maintains proximity, independently from its valence. It may be that an open confrontation affects the relationship less than a silent withdrawal (no conflict, where distancing equals cohesion) or an explicit suspension of being together (interactions break): In the first case, distancing equalizes cohesion and in the second case, exceeds it.

The final analysis shows precisely this: It is not the capacity of recognizing and depicting the core dimensions of friendship in ordinary times, as much as the capacity of recognizing high cohesion in difficult moments that protect from physical and verbal aggressiveness. This implies the awareness of being friends in spite of the risk of falling into quarrels or fights; the inability to recognize these risks and/or accept their occasional occurrence that deprives children of the social competence required to control aggressiveness in their social life.

We recognized that this study has several limitations. The unexpectedly high number of children conceiving distress as a non-conflictual situation prevented us from verifying how cohesion and distancing vary by gender within the various types of conflict categories. The reasons for choosing each specific representation have been derived conceptually *post hoc*, and in the absence of children’s explanations, they remain hypothetical. The representation of distress in the no-conflict drawings would have required a quantitative analysis of other indices, which were not the focus of this study. All these aspects could be addressed by further research, but we felt that the correlation between cohesion indices in distress drawings and the reduced aggressiveness is a result of a certain theoretical and practical meaning. In fact, it is an indication that the capacity of friendship maintenance represents a protective factor, easy to assess in young children with the pictorial task, and it shows how important it is, at the educational level, helping children to become aware of their way of acting with friends in times of difficulty. It seems that the awareness of what can upset the friendship, more than the individual negative actions which everyone can incur, is one of the factors that help to reduce aggressive behavior with peers.

## Data Availability Statement

The raw data supporting the conclusions of this article will be made available by the authors, without undue reservation.

## Ethics Statement

The studies involving human participants were reviewed and approved by Ethics committee of Department of Developmental and Socialization Psychology, Sapienza University of Roma. Written informed consent to participate in this study was provided by the participants’ legal guardian/next of kin.

## Author Contributions

AD selected the appropriate statistical approach and performed the statistical analysis. AB wrote the first draft of the manuscript. All authors contributed to the conception, design of the study and contributed to manuscript revision, and read and approved the submitted version.

## Conflict of Interest

The authors declare that the research was conducted in the absence of any commercial or financial relationships that could be construed as a potential conflict of interest.

## Publisher’s Note

All claims expressed in this article are solely those of the authors and do not necessarily represent those of their affiliated organizations, or those of the publisher, the editors and the reviewers. Any product that may be evaluated in this article, or claim that may be made by its manufacturer, is not guaranteed or endorsed by the publisher.
